# Quantized correlation coefficient for measuring reproducibility of ChIP-chip data

**DOI:** 10.1186/1471-2105-11-399

**Published:** 2010-07-27

**Authors:** Shouyong Peng, Mitzi I Kuroda, Peter J Park

**Affiliations:** 1 Department of Medicine, Brigham and Women's Hospital, Boston, MA, 02115 USA; 2 Department of Genetics, Harvard Medical School, Boston, MA, 02115 USA; 3 HST Informatics Program at Children's Hospital, Boston, MA, 02115 USA; 4 Center for Biomedical Informatics, Harvard Medical School, Boston, MA 02115 USA

## Abstract

**Background:**

Chromatin immunoprecipitation followed by microarray hybridization (ChIP-chip) is used to study protein-DNA interactions and histone modifications on a genome-scale. To ensure data quality, these experiments are usually performed in replicates, and a correlation coefficient between replicates is used often to assess reproducibility. However, the correlation coefficient can be misleading because it is affected not only by the reproducibility of the signal but also by the amount of binding signal present in the data.

**Results:**

We develop the Quantized correlation coefficient (QCC) that is much less dependent on the amount of signal. This involves discretization of data into set of quantiles (quantization), a merging procedure to group the background probes, and recalculation of the Pearson correlation coefficient. This procedure reduces the influence of the background noise on the statistic, which then properly focuses more on the reproducibility of the signal. The performance of this procedure is tested in both simulated and real ChIP-chip data. For replicates with different levels of enrichment over background and coverage, we find that QCC reflects reproducibility more accurately and is more robust than the standard Pearson or Spearman correlation coefficients. The quantization and the merging procedure can also suggest a proper quantile threshold for separating signal from background for further analysis.

**Conclusions:**

To measure reproducibility of ChIP-chip data correctly, a correlation coefficient that is robust to the amount of signal present should be used. QCC is one such measure. The QCC statistic can also be applied in a variety of other contexts for measuring reproducibility, including analysis of array CGH data for DNA copy number and gene expression data.

## Background

Chromatin immunoprecipitation on tiling arrays (ChIP-chip) has been widely used to study genome-wide binding sites of transcription factors [1-5] and other protein complexes [6,7], as well as histone modifications [8]. To ensure data quality, ChIP-chip experiments are generally performed in replicates (two or three), and their reproducibility is used to determine whether the data are of sufficient quality and whether further experiments or validations are necessary. A number of factors impact the reproducibility of ChIP-chip data, including uniformity of cross-linking and sonication, specificity of the antibody, efficiency of chromatin immunoprecipitation, quality of the probes, and biological variability.

**Figure 1:**
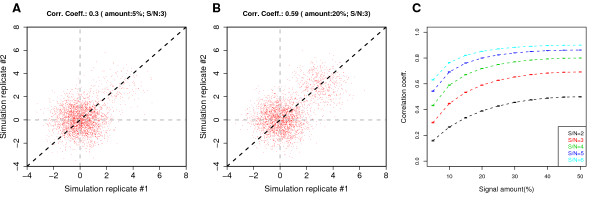
**The PCC is not a robust statistic for measuring reproducibility of ChIP-chip data**. (A) A scatterplot between probe signals of two simulated ChIP-chip replicates. The total number of probes is 300,000; 5% of the probes are assumed to have binding signal, with signal-to-noise ratio (SNR) of 3. Gaussian noise with standard deviation s.d.= 1 was added to all the probes for each simulated replicate. The PCC is 0.3. (B) Same as (A) but with 20% of the probes displaying binding signal. The PCC nearly doubles to 0.59. (C) The PCC at the probe level depends on the amount of binding for range of SNRs. Each point is an average over 100 simulated replicate pairs; error bars represent a 95% confidence interval. Although the PCC correctly reflects the data quality as defined by SNR, it underestimates substantially when the amount of binding signal is low for any fixed SNR.

To assess the reproducibility of ChIP-chip data, a correlation coefficient is most often used to compare the replicates at the probe level [9]. The Pearson correlation coefficient (PCC) is the most common, but the non-parametric Spearman or Kendall correlation coefficient can also be used. However, while the simplicity of the standard correlation coefficient is appealing, it suffers from a serious problem: the statistic depends not only on the reproducibility of the data but also on the amount of DNA-protein binding or post-translational modification (henceforth collectively referred to as "signal") present in that experiment, as we will demonstrate (See Fig. 1). That is, when a large amount of signal is present, the correlation coefficient tends to be higher than when a small amount of signal is present. In the latter case, the background signal contributes significantly and leads to a lower coefficient, even though PCC is generally sensitive to the outlying values. This lack of robustness to the amount of signal is an undesirable property, as it means that correlation coefficients cannot be properly compared between different experiments, except in the likely event that the amount of binding is exactly the same. A PCC of 0.7 in one experiment does not indicate the same data quality or reproducibility as 0.7 in another experiment if the amount of binding is different between the two experiments. This phenomenon is especially true for two-color ChIP-chip experiments, in which log-ratios rather than absolute intensities are used to measure correlation. When correlation is computed on the absolute intensities, the low values contribute relatively little to the statistic because their variability is small compared to the range of intensities. When a control channel or experiment is used to generate log-ratios, however, these background probes have large variability around zero and impact the correlation substantially.

Another way to compare replicates is after further processing of the data, after regions (or "clusters") of binding or modification are identified. While we have often employed this approach in our previous analysis [6], this is not a simple process. For instance, the noise level in each replicate may be different and so the thresholds for identification of enriched regions must be defined in each case. Depending on how these thresholds are defined, the results can vary substantially. Furthermore, the agreement among the identified clusters is difficult to define precisely. One can measure, for instance, the proportion of enriched clusters that overlap above a given percentage, at the expense of introducing another parameter. But, other complications also arise, such as a cluster in one experiment that encompasses multiple clusters on another array, making it unclear how to best compute the proportion. One can also choose to measure the fraction of overlap in base-pairs, but this quantity becomes biased in favor of data sets that have broad clusters (e.g., certain histone modifications) rather than sharp marks (e.g., transcription factors). These complications may be avoided in many situations if a more robust criterion for data quality and reproducibility exists. In the end, however, neither point-wise nor cluster-wise comparison is perfect; the overall decision on the quality should depend on several statistics including these two.

In this work, we propose the Quantized correlation coefficient (QCC) that is more robust to the amount of signal present. We first use simulated data to measure the extent to which the PCC is influenced by the amount of signal in the data (how much of the genome is covered). This is done at varying levels of enrichment over background, which we refer to as the signal-to-noise ratio (SNR). Then we describe QCC and compare it to the PCC. We also describe the methodological issues for this statistic and use real data to further demonstrate its usefulness.

## Results and Discussion

### Quantized correlation coefficient (QCC)

The Pearson correlation coefficient between two vectors, (*x_i_, y_i_*), *i *= 1, ..., *n*,(1)

fails to be robust at different signal amounts because the background noise can have substantial influence on the statistic when the amount of binding is low. The main idea behind the proposed quantization and merging procedure is first to identify the probes containing background noise with an iterative step and then to reduce their impact on the correlation coefficient. This involves quantization-the process of constraining a continuous set of values by a discrete set-and hence the new statistic is called the Quantized correlation coefficient.

1. *Initial quantization: *For each data set, all the probe-level data are binned into *B*_0 _groups of equal size (number of probes) based on the signal quantiles, starting with 1 for the probes with smallest intensities. The probes in each group are assigned as their new values integers 1, 2, ..., *B*_0 _that correspond to their group number. For instance, with *B*_0 _= 10, all probes that were in the 3rd decile in terms of intensity now have the new values 3. *B*_0 _is a user-specified, initial number of bins for quantization. The choice of this parameter is discussed later.

2. *Merging of two neighboring groups: *Given *B *bins, There are *B *- 1 possible ways to merge two consecutive bins. For each possible merging, reassign the probes with 1, 2, ..., *B *- 1 based on the new group configurations and calculate their corresponding PCC. After PCC is computed for every *B *- 1 possible configurations, choose the merging that most improves the correlation, and update the group assignment and probe values.

3. *Loop: *Repeat the above merging procedure until the correlation coefficient no longer improves. This defines the final groupings and values of the data points on which the correlation is computed.

In part, this method resembles the Spearman correlation coefficient, which is the PCCs applied on the ranks of the original data. Because the correlation is computed on the ranks, QCC assesses how well an arbitrary monotonic function captures the relationship between the two replicates without any distributional assumptions. It also assumes that the consecutive ranks are equidistant (before quantization) and thus suppresses the effect of outliers in the data. On the other hand, whereas the Spearman correlation utilize the ranks of all data points equally in the computation, QCC collapses the the noisy part of the data (random data points near the origin corresponding to unbound probes) and reduces its contribution.

### Analysis of simulated ChIP-chip data

We first study the impact of signal amount on the PCC. We generated synthetic ChIP-chip data with 300,000 total number of probes (close to the number of probes on the NimbleGen tiling arrays we have used previously [6,10]), with the probes divided into background and signal groups. We assume that the SNR is fixed for each simulation. Gaussian noise is added to each measurement and, without loss of generality, the standard deviation is set to 1. Thus, signal coverage (i.e. percentage of probes carrying binding signals) and signal intensity relative to noise level (i.e. signal-to-noise ratio) are the only two parameters for each ChIP-chip simulation.

Fig. 1A and 1B show two simulated sets of experiments with two replicates each, generated with the same SNR but with different amounts of binding signal (5% and 20%, respectively). Because they have the same SNR, an ideal statistic for reproducibility should be the same in both cases. However, the PCCs are 0.3 and 0.59, respectively, showing that the PCC depends heavily on signal coverage. Fig. 1C shows the correlation coefficients at varying amounts of signal, for five different SNRs. The correlation coefficient correctly orders the experiments according to their SNRs at a given signal amount. But within a single SNR, it varies substantially. How this fluctuation can lead to misleading assessment of reproducibility is clear. The two replicates that have SNR of 4 and coverage of 15%, for instance, have a higher correlation coefficient between them than the two replicates with SNR of 6 but coverage of 5%. The coefficients do plateau after 30% or so, but the signal amount in most real experiments are much smaller. For instance, even if a transcription factor binds at 30,000 places in the human genome with 1 kb resolution of binding on the array, this corresponds to only 1%.

**Figure 2:**
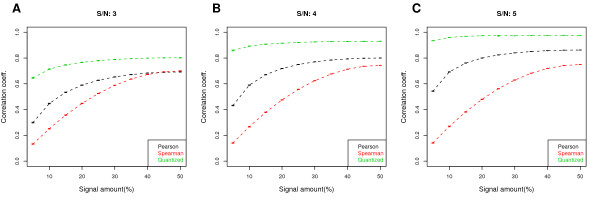
**Improved robustness in the QCC in simulated ChIP-chip data**. A comparison of different correlation coefficients (the Pearson, the Spearman, and the Quantized) as a function of signal coverage, for SNR values of 3, 4, and 5, respectively for (A), (B), and (C). The number of initial bins is 100. The QCC is less sensitive to signal coverage as the nearly flat profile indicates, but it is still sensitive to data quality (i.e., the SNR in the simulated data). Each point is an average over 100 simulated replicate pairs; error bars represent a 95% confidence interval.

We applied our quantization and merging method to see whether the new coefficient is more stable. In Fig. 2, we compare QCC with the Pearson and Spearman correlation coefficients in the simulated ChIP-chip replicate data at different SNRs. In all three cases, although QCC (green) is not completely flat, it is substantially more robust across different signal coverages. For SNR of 3 (Fig. 2A), the Pearson and Spearman correlation coefficients fluctuate by a factor of 2 and 3, respectively, while QCC changes by 10-20%. For higher SNR, QCC is even more stable. Although it is not possible to determine whether one value of correlation is more correct or less biased than another, QCC is more robust to the signal amount. It also appears to reflect the quality of the data more accurately: when the signal coverage is low for a given SNR, noise in the background prevails in PCC but has less impact for QCC. That QCC is consistently higher than the other two does not indicate that it is less biased, but QCC is closer to the coefficient one would obtain from the signal part of the data (an example is given in the next section). In addition, QCC is still sensitive to data quality and increases with higher SNR. This is the case for the Pearson coefficient but not for the Spearman coefficient.

**Figure 3:**
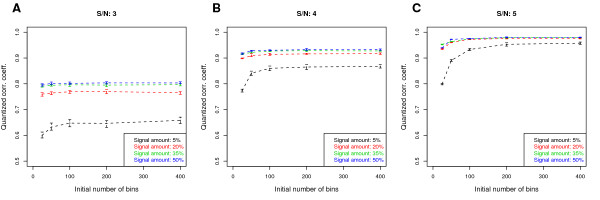
**Effect of initial number of bins on the QCC**. (A), (B) and (C) correspond to different signal intensities (SNR = 3, 4, and 5). QCC is robust when initial number of bins is large enough (above 100 or 200), even when a large amount of signal is present. Each point is an average over 100 simulated replicate pairs; error bars represent a 95% confidence interval.

As the initial number of probe bins is the only parameter we need to specify in the quantization and merging procedure, we investigate its effect on QCC. Fig. 3 shows the results, which suggest that QCC quickly stabilizes when initial number of bins is large enough, e.g., 100. We have used 100 bins in the simulations above based on this result. The only exception would be for the cases with very high SNRs and low coverage, but the computation is fast and a larger bin number can be easily used.

### Analysis of MSL complex ChIP-chip data

Dosage compensation is a process in which organisms attempt to equalize X-linked gene expression when the X-chromosome copy numbers are different between males and females. Without this process, gene products between X and autosomes would not be balanced in each sex. In mammals, this is achieved by X-inactivation, which silences gene expression from one of the two X chromosomes in females. In *Drosophila*, the opposite strategy of up-regulating the single X chromosome by two-fold is employed. How the entire male X chromosome is regulated on such a large scale is a fascinating question, which we have studied extensively. While some aspects of this process are still not clear, a key step involves the *Drosophila *male-specific lethal (MSL) complex. Previously, we have used custom NimbleGen tiling arrays (~50-mer, 100-bp spacing) to examine the binding of this complex on the X chromosome as well as on an autosome of similar size (2L) that serves as an internal control. Our experiments and analysis identified precise locations on the X chromosome on which the MSL complex binds to start the process of up-regulation of the X-linked genes [6,11].

**Figure 4:**
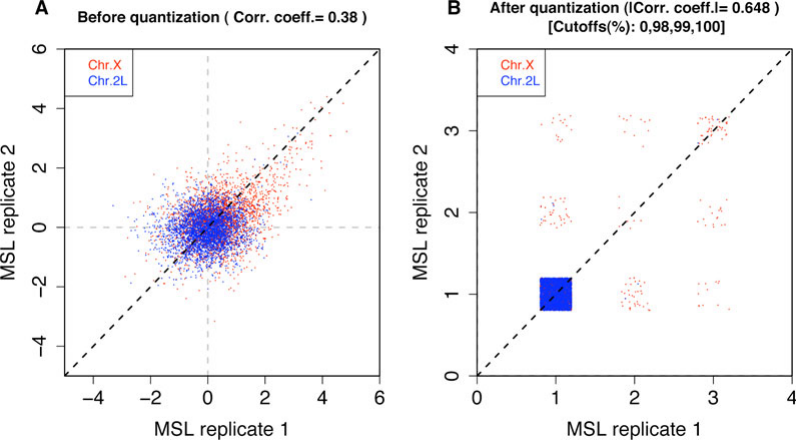
**Scatterplots before and after quantization**. Quantization and merging identifies background signals and suppresses their effect on the reproducibility measure, as shown in the case of MSL complex binding in *Drosophila*. (A) A scatterplot between replicates shows that the data quality of the replicates is fairly good, as almost all signal probes are from the X chromosome (red) rather than an autosome (blue) for the complex that is known to have X-specific binding [13]. The standard PCC at the probe level is 0.38, as the background signal makes a substantial contribution to the statistic. (B) The final configuration (with jittering to view data points that would otherwise be overlapping) of the quantization and merging procedure with 100 as the initial number of bins. The cut-offs are the quantiles to which the iteration converges. Almost all the probes from the autosome are grouped into the background as expected, and the correlation of the ranked data at the probe level increases to 0.65.

The specific binding of the complex on the X chromosome makes this data set an ideal one for studying methodological questions in ChIP-chip data analysis, as we know that all binding signals must come from the X chromosome [12]. In Fig. 4A, the scatterplot between two ChIP-chip replicates is shown for the binding of the MSL complex (a mutant form of the complex that also has X-specific binding but at fewer sites, as described in [13]). Fig. 4A shows the probe values on the X chromosome and the autosome in different colors. It appears that nearly all the high signals come from red points corresponding to the X chromosome and that they correlate fairly well. Profiles along the chromosomal positions also indicate that the binding sites are well reproduced in this data set. However, the standard PCC at the probe level is only 0.38. Although there is no consensus definition of what a 'high' or 'low' correlation coefficient is, 0.38 appears to be lower than what one may expect from the signal part of the data. This happens because the binding signal in this data set is very small (about 2%) as in most ChIP-chip data and the background noise dominates in the computation of the coefficient.

We applied our proposed quantization and merging method with 100 initial number of bins to this data set. Fig. 4B shows the final configuration (shown with random perturbations, sometimes known as jittering, to view the data points that would be overlapping otherwise). As expected, almost all the probes from the 2L autosome are grouped into the background part, and the X-specific signal probes are grouped separately. The resulting correlation increases from 0.38 to 0.65. We believe this is a better indicator for these ChIP-chip data quality and reproducibility. When we compute the PCC based only on the intersection of the top 10% probes from each replicate, it is 0.69, revealing that the signal portion is well correlated between replicates and that the reproducibility measured by QCC is a reasonable estimate. That QCC is not simply a blind inflation of correlation coefficient can be examined by the QCC for mock data. When we test QCC for the mock data, which were available only for the wildtype MSL complex [6], the QCC is 0.38 while the PCC is 0.37 (for this data set, the PCC and QCC for the IP experiments were 0.82 and 0.95, respectively).

### Comparison to cluster-level reproducibility

We have already described in the introduction some of the complications that arise if reproducibility is measured at the cluster level, including how to identify clusters accurately and how to measure overlap between two sets of clusters. Nonetheless, we carry out one direct comparison between computing reproducibility at the probe level versus at the cluster level, both using simulated data and real data, to get a rough idea between the two types of comparisons.

**Figure 5:**
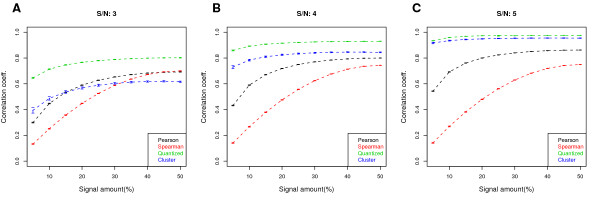
**Cluster-level reproducibility in simulated ChIP- chip data**. We superimpose the reproducibility measured at the cluster-level (blue line) on Fig. 2. The cluster-level reproducibility is relatively insensitive to the amount of binding in the data, similar to what is observed for QCC. See the text for details of the simulation.

Numerous peak callers (e.g., see [14]) have been proposed and their performance varies widely depending on the type of binding profiles (e.g., sharp versus broad) and platforms. For our simulated data, we set the size of every cluster as 5 probes and add noise of varying magnitude to each probe, for a variable number of clusters. Then a region spanning a minimum of 4 consecutive probes (*~*150bp for probes with 50-bp median spacing) above a quantile threshold is then called as a cluster. This very simple simulation ensures that the peak calling is done accurately and there is little variation coming from that step. To determine concordance, the probes identified as part of a peak is replaced with 1 and the rest are replaced with 0, and the Pearson correlation coefficient is computed between the replicates. In Fig. 5, the results of this simulation is shown on top of the plots shown in Fig. 2. We see that the cluster-level reproducibility, if the clusters can be accurately identified, has a desirable property of not being strongly influenced by the amount of binding signal. This can be seen by the blue lines that are relatively flat, similar to the green QCC lines, at varying signal-to-noise levels. We also see that cluster-level reproducibility is more sensitive in that it has a greater range of values for the three signal-to-noise ratios.

**Figure 6:**
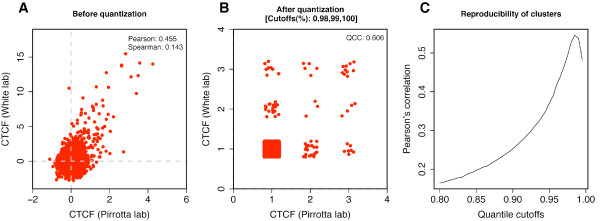
**Cluster-level reproducibility of data from different laboratories**. Two laboratories independently produced ChIP-chip data for CTCF in Drosophila S2 cells. (A) A scatterplot between the two datasets for the probes on the X chromosome (only 3000 points are shown for legibility). (B) After quantization (with jittering to view data points that would otherwise be overlapping). (C) Cluster-level concordance at varying quantile thresholds. The maximum of the cluster-level correlations is 0.546.

For real data, we compared CTCF ChIP-chip data in *Drosophila *S2 cells produced in two laboratories (the Pirrotta lab and the White lab), as part of the model organism Encyclopedia of DNA Elements (modENCODE) project http://intermine.modencode.org. Fig. 6A shows the scatterplot between the two datasets for the probes on the X chromosome and Fig. 6B shows the quantized view with jittering, as was done in Fig. 4B. The PCC is 0.455 while the QCC is 0.606. We computed cluster-level concordance at varying quantile thresholds (Fig. 6C) and the maximum correlation was 0.546, which was a little closer to QCC than to PCC.

Overall, the probe-level and cluster-level reproducibility measures are complementary and both types of analysis can assist in determine the data quality. QCC is much simpler to compute and is not burdened with the dependence on the quality of the peak-calling algorithm or the threshold (generally the false discovery rate or FDR) used to define peaks. For instance, a low FDR will select only the most prominent peaks, which tend to be quite reproducible; a higher FDR will result in inclusion of less prominent, lower enrichment peaks that are less reproducible. Thus, for many data sets, the user will get a different impression on the quality of the data, depending on the FDR value used. On the other hand, any subsequent analysis of ChIP-chip data will involve characterization of the peaks, such as searching for a shared motif or co-binding patterns with other factors. Thus, it is reasonable to consider the reproducibility of data at the cluster-level.

## Conclusions

We have shown that standard correlation coefficients do not properly reflect the agreement between ChIP-chip replicates, especially when amount of binding is low. Our proposed procedure groups the background probes and suppresses their contribution to the correlation so that reproducibility can be better captured. This procedure requires no *a priori *knowledge about the features, such as enrichment/depletion, high/low coverage, and noise level. The initial number of bins is the only parameter involved but we have shown that the results are stable beyond a sufficiently large number.

As mentioned in Background, there are other ways to measure reproducibility in ChIP-chip data. In particular, a correlation analysis approach discussed here completely ignores the signal locations, and accounting for this by comparing replicates after identification of enriched regions is an alternate approach that should be done at the same time. Other essential steps in quality control are to visualize the data in a genome browser and to compare the enrichment at specific loci with qPCR data. Nonetheless, a simple correlation analysis is often done in practice and it is therefore important to address the pitfalls in this approach. Again, we do not claim that QCC is more accurate than PCC for a given set of data. Such a statement is difficult to make in general and the maximization steps we take could be overcompensating the PCC estimate. Instead, we claim that it has the desirable property of robustness against the varying fraction of signal we observe in real experiments and that it accurately reflects SNR in the data.

QCC was developed in the context of ChIP-chip data, but it can be directly applied to other applications in which estimation of the similarity or reproducibility between any two sets of measurements is needed, as long as data can be ordered and binned into subgroups. As illustrated in this paper, QCC is most useful when a large part of the data consists of background noise and its amount is a confounding factor in interpretation of the standard correlation coefficients. In genome-wide array measurements, this occurs frequently. In many gene expression studies, the majority of the genes may not be expressed or expressed at low levels; in many array comparative genomic hybridizations (CGH), only a small fraction of the genomic DNA may display copy number aberrations. Quality control of these experiments requires a consistent measure of reproducibility of replicate experiments and QCC is one possible measure.

## Methods

### Computational efficiency

The iterative merging of neighboring bins implies that there are *B *- 1 possibilities to consider at each step with *B *bins, and so the upper bound for the total number of possible combinations in the procedure is *B*_0_(*B*_0 _- 1)/2. This method is a local search algorithm, as the two bins that are combined at one step cannot be separated again. A global search can be contemplated, but given that most merging occurs near zero where the noisy data are concentrated, it is unlikely to result in any significant improvement.

### Comparison to other robust correlations

Other correlation measures have been proposed previously in the statistics literature. The most applicable one to the current context is an iterative optimization procedure called alternating conditional expectation (ACE), developed by Breiman and Friedman [15]. This was developed in the regression analysis setting in which nonlinear transformation are sought to produce the best-fitting additive model. Let the random variables *X*_1_, ..., *X_p _*be predictors and *Y *the response variable. The idea behind this method is to find optimal mean-zero transformation function *θ*(*Y*), *ϕ*_1_(*X_i_*), ..., *ϕ_p_*(*X_p_*) that minimizes the fraction of variance not explained in a regression of *θ*(*Y *) on :

The ACE procedure uses only bivariate conditional expectations and converges to an optimal solution. When only a single predictor is used, estimation of the optimal transformation becomes a method for estimation of maximal correlation between two random variables. In genomics, this was utilized previously as a basis for normalization scheme called simultaneous ACE, which maximizes correlation between replicate array experiments [16].

The concept of correlation is such that it is difficult to determine whether one estimate is superior to another in a given problem, except in few extreme cases. For QCC, we present evidence that it is robust with respect to the signal amount and increases as the signal-to-noise ratio gets larger (Fig. 2), but we cannot prove that the absolute numerical value is more correct. In our assessment, the main advantage of ACE is that it has a precise formulation and a more theoretical framework. QCC, on the other hand, is more intuitive in our view and is a greedy algorithm, yet it appears to work in a desired manner.

## Authors' contributions

SP designed the method with input from MIK and PJP. SP and PJP wrote the manuscript. All have read and approved the final manuscript.
